# The recent escalation in strength of pyrethroid resistance in *Anopheles coluzzi* in West Africa is linked to increased expression of multiple gene families

**DOI:** 10.1186/s12864-015-1342-6

**Published:** 2015-03-01

**Authors:** Kobié H Toé, Sagnon N’Falé, Roch K Dabiré, Hilary Ranson, Christopher M Jones

**Affiliations:** Department of Vector Biology, Liverpool School of Tropical Medicine, Liverpool, L3 5QA UK; Centre National de Recherche et de la Formation sur le Paludisme, Ouagadougou, 01BP 2208 Burkina Faso; Institut de Recherche en Sciences de la Santé/Centre Muraz, Bobo-Dioulasso, 01 BP 545 Burkina Faso

**Keywords:** Anopheles coluzzi, Pyrethroid resistance, Detoxification enzymes, Transcriptomics, Vector control

## Abstract

**Background:**

Since 2011, the level of pyrethroid resistance in the major malaria mosquito, *Anopheles coluzzi*, has increased to such an extent in Burkina Faso that none of the long lasting insecticide treated nets (LLINs) currently in use throughout the country kill the local mosquito vectors. We investigated whether this observed increase was associated with transcriptional changes in field-caught *Anopheles coluzzi* using two independent whole-genome microarray studies, performed in 2011 and 2012.

**Results:**

Mosquitoes were collected from south-west Burkina Faso in 2011 and 2012 and insecticide exposed or non-exposed insects were compared to laboratory susceptible colonies using whole-genome microarrays. Using a stringent filtering process we identified 136 genes, including the well-studied detoxification enzymes (p450 monoxygenases and esterases) and non-detoxification genes (e.g. cell transporters and cuticular components), associated with pyrethroid resistance, whose basal expression level increased during the timeframe of the study. A subset of these were validated by qPCR using samples from two study sites, collected over 3 years and marked increases in expression were observed each year. We hypothesise that these genes are contributing to this rapidly increasing resistance phenotype in *An. coluzzi*. A comprehensive analysis of the knockdown resistance (*kdr*) mutations (*L1014S*, *L1014F* and *N1575Y*) revealed that the majority of the resistance phenotype is not explained by target-site modifications.

**Conclusions:**

Our data indicate that the recent and rapid increase in pyrethroid resistance observed in south-west Burkina Faso is associated with gene expression profiles described here. Over a third of these candidates are also overexpressed in multiple pyrethroid resistant populations of *An. coluzzi* from neighbouring Côte d’Ivoire. This suite of molecular markers can be used to track the spread of the extreme pyrethroid resistance phenotype that is sweeping through West Africa and to determine the functional basis of this trait.

**Electronic supplementary material:**

The online version of this article (doi:10.1186/s12864-015-1342-6) contains supplementary material, which is available to authorized users.

## Background

Resistance to the pyrethroid insecticides in *Anopheles* malaria mosquito vectors is now widespread throughout Sub-Saharan Africa [1]. The recent gains in reducing the burden of malaria, achieved largely by the scale-up of long lasting insecticide treated nets (LLINs), are under threat as pyrethroids represent the only insecticide class approved for use on LLINs.

The south-west of Burkina Faso was one of the first regions to report pyrethroid resistance in the local malaria vector population [2]. Since the late 1990s the level of resistance has gradually increased with the intensive agricultural activity in the area a likely contributing factor [3,4]. A recent survey of resistance in the village of Vallée du Kou in the south-west of the country, conducted between 2011 and 2013, has highlighted the scale of the problem with resistance levels of over 1000-fold described [5]. Laboratory assays found that none of the LLINs currently used throughout the country gave acceptable levels of mortality against local mosquitoes, raising serious concerns over the efficacy of current vector control strategies in the country.

The main resistance mechanisms to pyrethroids include target site mutations in the voltage sodium channel (the knockdown resistance mutations (*kdr*), *L1014F*, *L1014S* and *N1575Y*) and metabolic resistance including the over-expression of the three major detoxification enzyme families (p450 monooxygenases (p450s), glutathione-S-transferases (GSTs) and the carboxylesterases) (reviewed in [6]). While the rise and spread of the *kdr* alleles in *Anopheles gambiae* throughout Sub-Saharan African are undoubtedly associated with DDT and pyrethroid resistance, they do not account for all of the variation in the phenotype [7,8].

The design of microarray-based platforms for characterising gene expression profiles in *Anopheles gambiae*, initially based on detoxification enzymes [9], and more recently, using whole-genome wide arrays [10], has provided evidence for specific genes contributing to insecticide resistance in wild-caught *An. gambiae* (e.g. [11-13]). Commonly over-expressed genes have been identified in independent studies across Sub-Saharan Africa including, for example, specific P450 enzymes (e.g. *CYP6M2* and *CYP6P3*) which have been shown to metabolize pyrethroids and other insecticide classes [10,12,14-16]. Most of these transcription studies conducted to date have compared resistant mosquitoes, collected at a single time point, to either sympatric non-exposed insects or laboratory susceptible strains. Few studies have followed transcriptional changes in wild-caught *An. gambiae*, from a single origin, displaying increasingly high levels of resistance at the whole-genome level.

We followed the rapid rise of deltamethrin resistance in the village of Vallée du Kou 7 (VK7) and collected mosquitoes either exposed or non-exposed to deltamethrin in 2011, 2012 and 2013 for transcriptional analysis against laboratory susceptible strains. Using a filtering process based on the over-expression of candidates in VK7, as well as an increase in expression from 2011 to 2012, we generated a list of candidate genes, including well-studied detoxification enzymes, transporters and cuticular genes that are likely contributing to the exceptionally strong pyrethroid resistance phenotype in this population. The investigation also highlighted that target site resistance, whilst highly prevalent, was not strongly associated with survival after pyrethroid exposure.

## Results

### Deltamethrin resistance associated gene expression

**Figure 1:**
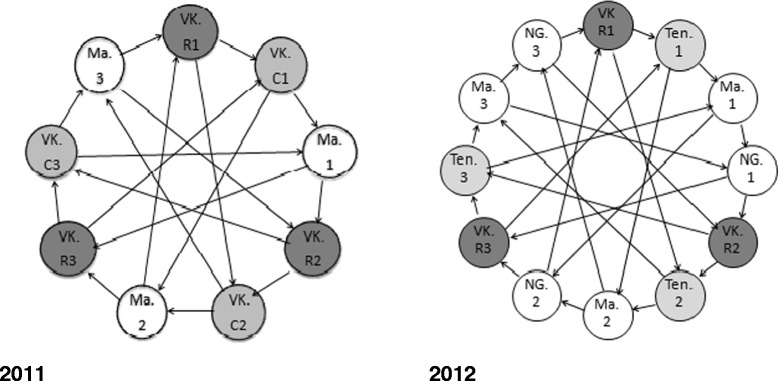
**Interwoven loop designs for microarray experiments performed in 2011 and 2012.** In 2011, deltamethrin selected mosquitoes from VK7 (VKR; LT_50_ = 254 min), unexposed mosquitoes from VK7 (VKC) and the Mali susceptible lab strain (MAL) were compared. In 2012, four mosquitoes populations were compared including deltamethrin selected mosquitoes from VK7 (VKR; 600 mins exposure), unexposed mosquitoes from a deltamethrin resistant population approximately 120 km from VK7, Tengrela (TEN), the susceptible MAL strain and a second fully susceptible lab strain from N’Gousso, Cameroon (NG). The direction of the arrows represents a cy3 to cy5 hybridisation and three biological replicates were used for each comparison.

**Figure 2:**
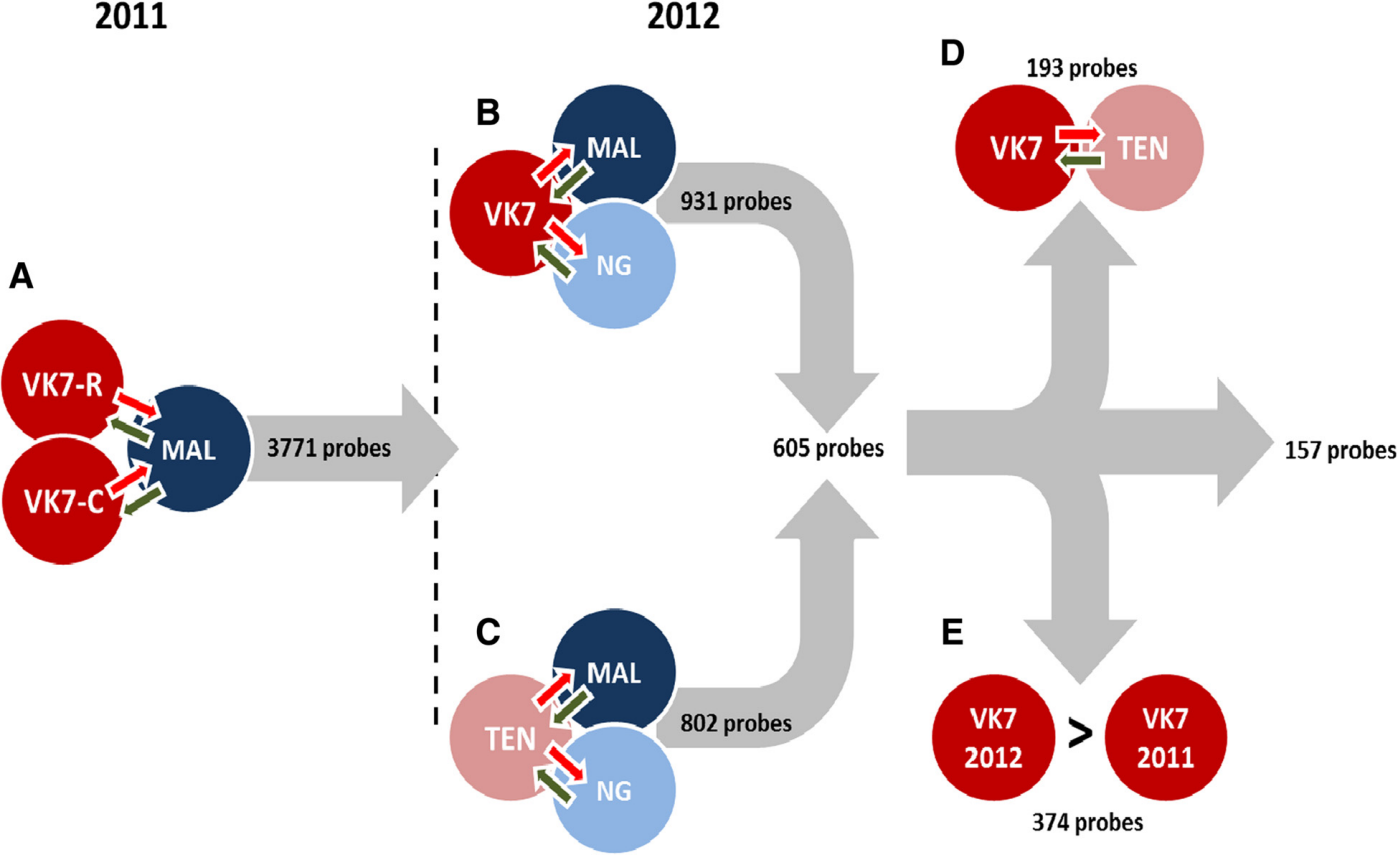
**Microarray data analysis schema showing the different steps and the number of probes obtained after each filtering step.** Each filtering step is based on our hypothesis of over-expression in field resistant populations compared to laboratory susceptible strains (steps A, B and C), over-expression in VK7 compared to TEN (step D) and up-regulation in 2012 compared to 2011 according to the VK7-MAL comparison (step E). The green and red arrows represent dye swaps between the samples.

Two whole-transcriptome microarray experiments were performed on VK7 *An. coluzzi* collected as part of June 2011 and July 2012 bioassays (described in [5]) to identify candidate genes associated with deltamethrin resistance. This included mosquitoes that had survived more than 10 hours exposure to the diagnostic dose of pyrethroids, an unprecedented high level of resistance for malaria vectors. A detailed experimental design is given in Figure 1. In 2012, we expanded the study to include a second susceptible strain and an additional field strain (Tengrela; TEN) with a less intense resistance phenotype (~50% mortality to deltamethrin in a one-hour WHO diagnostic dose test) to improve confidence in our candidate gene list. To identify candidate genes in VK7 we made three underlying assumptions: a) candidate resistance genes are more highly expressed in resistant field populations versus susceptible lab colonies (MAL originating from Mali & NG originating from Cameroon), b) the same underlying mechanisms were responsible for resistance in 2011 and 2012 and c) genes conferring pyrethroid resistance would be more highly over-expressed in VK7 compared to TEN. A detailed analysis schema is provided in Figure 2 and the Methods.

### Genes over-expressed in resistant field populations

The filtering approach which compared all field resistant populations to the susceptible laboratory strains, using data from both 2011 and 2012, left 605 probes (representing 487 genes) (Additional file 1) including 15 cytochrome P450s, 9 glutathione S-transferases (GSTs), 3 carboxylesterases and many other non–detoxification genes. The top five over-expressed detoxification genes based on the VK7/MAL comparison in 2012 included *GSTE2* (AGAP009194, four probes, average FC = 9.48), *CYP4G16* (AGAP001076, four transcripts, average FC = 6.30), *GSTS1*_1 (AGAP010404, three probes, average FC = 3.7), *CYP9J5* (AGAP012296, FC = 3.03) and *CYP6P1* (AGAP002868, four probes, average FC = 2.69). The most over-expressed non-detoxification genes consisted of an ATP synthase (AGAP006879, FC = 22.30), glycoside hydrolase (AGAP009110, FC = 9.51), a cuticular protein with chitin binding domains *CPAP3-A1b* (AGAP000987, FC = 9.35) and a takeout protein associated with insect circadian clocks (AGAP004262, FC = 11.48). Additional over-expressed genes, which were also up-regulated in later filtering steps (see below), included an aquaporin (AGAP010326, FC = 7.97), chymotrypsin-1 (AGAP006709, FC = 5.18) and the carboxylesterase, *COEAE3G* (AGAP006724-RA, FC = 2.56, four probes) (Additional file 1).

### Genes expressed at higher levels in deltamethrin survivors from 2012 compared to 2011

Out of the 605 probes over-expressed in all the field resistant populations compared to the laboratory susceptible strains, 374 probes had a higher fold change in 2012 than in 2011 based on the VK7/MAL comparison (Step E in Figure 2; Additional file 2). As resistance levels increased dramatically between these years we hypothesised that this probe list may include genes contributing to the resistance phenotype. The five GSTs genes (*GSTE2* (AGAP009194, FC = 9.48), *GSTMS3* (AGAP009946, FC = 2.52), *GSTS1*_2 (AF513639, FC = 2.42), *GSTM1* (AGAP000165, FC = 2.24), and *GSTE5* (AGAP009192, FC = 2.37)), five out the fifteen cytochrome P450s (*CYP4G16*, *CYP6P1*, *CYP9J5*, *CYP6Z3* (AGAP008217, FC = 2.36) and CYP9M1 (AGAP009363, FC = 2.27)) and two of out the three carboxylesterases (COEAE3G (AGAP006724, FC = 2.41) and *COEAE4G* (AGAP006725, FC = 1.37)) remained after this filtering approach. The highest fold-change differences between 2012 and 2011 were observed for genes already showing high constitutive expression including the chymotrypsin-1 (AGAP006709, FC = 3.64), aquaporin (AGAP010326, 2.60), cuticular protein *CPAP3-A1b* (AGAP000987, FC = 2.19) and the ATP synthase (AGAP006879, FC difference = 2.14). The P450, *CYP4G16* (AGAP001076), which has been implicated in pyrethroid resistance elsewhere [17], also showed an increase in expression in 2012.

**Figure 3:**
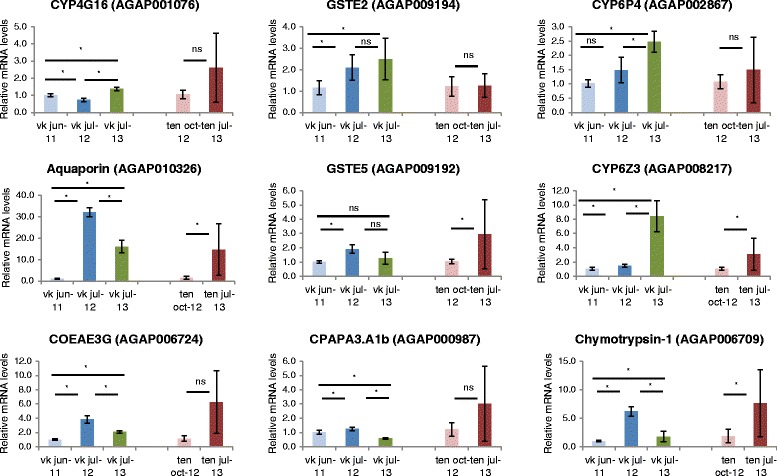
**Relative mRNA levels of candidate resistance genes measured by qPCR.** The level of expression was measured from *An. coluzzi* from Vallée du Kou (2011, 2012, and 2013) and Tengrela (2012 and 2013) in samples independent to those used in the microarray experiments and not exposed to insecticide. The relative values are presented according to the ddCt method [18] ± SEM of six biological replicates. A two-tailed Student’s *t*-test was performed between the yearly values within population (log-scale). *= p < 0.05; n.s. = not significant.

Seven of the nine candidate genes chosen for qPCR validation were expressed at higher levels in VK7 in 2012 compared to 2011 (two tailed *t*-test, *P* < 0.05) (Figure 3).

### Genes expressed at higher levels in VK7 compared to TEN

**Figure 4:**
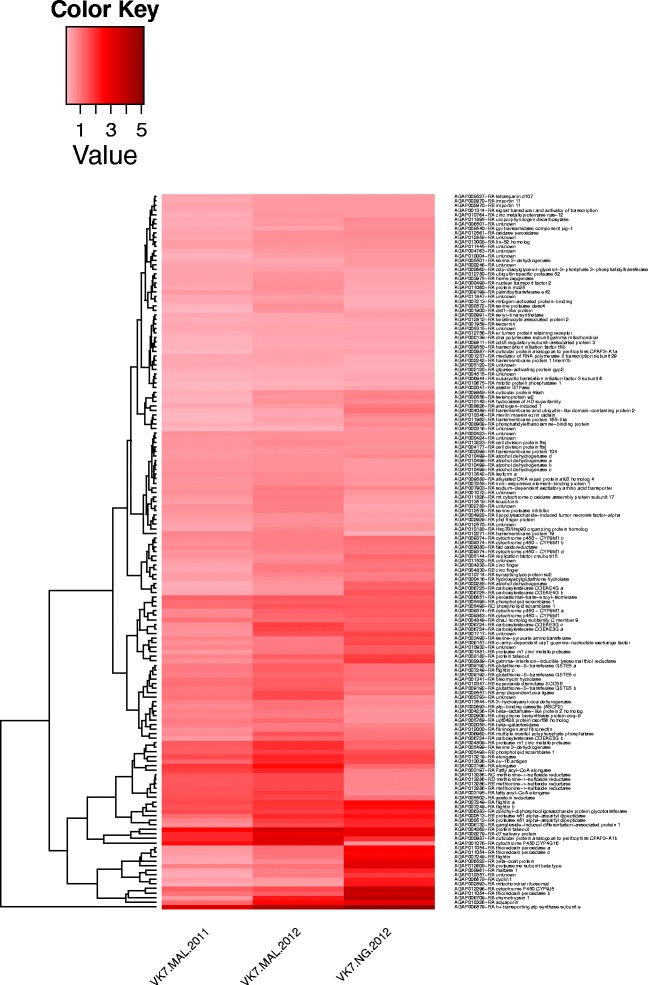
**Hierarchial clustering analysis of the candidate probes for deltamethrin resistance over-expressed in** ***An. coluzzi*** **from VK7.** Clustering was performed using the Euclidean distance method on the 157 probes which were over-expressed in VK7 versus MAL in 2011 and 2012 as well as those over-expressed against NG. The colour scale represents the log fold change of VK7 against the susceptible strain.

We included *An. coluzzi* mosquitoes from Tengrela (TEN) in the 2012 experiment to see whether a common set of genes were up-regulated in pyrethroid resistant mosquitoes from VK7 and TEN. Considering that resistance is higher in VK7 than TEN, we only retained probes significantly over-expressed in VK7 compared to TEN. This gave a final candidate gene list containing 157 probes (Additional file 3). A hierarchical clustering analysis based on the expression profiles of three comparisons between VK7/MAL (2011), VK7/MAL (2012) and VK7/NG (2012) is presented in Figure 4. If putative alternative transcripts of the same gene and duplicate probes are removed, the candidate gene list reduces further to 136 unique genes. Several of the detoxification genes having higher FC in 2012, including *CYP4G16*, *CYP9J5*, *CYP9M1*, *COEAE3G*, *GSTE5*, were retained in this list as well the components of the cuticle (e.g. *CPR 73*, *CPAPA3-A1a* and *CPAPA3-A1b*) and the chymotrypsin-1, aquaporin and ATP synthase (Additional file 3).

### Genes expressed at higher levels in TEN compared to VK7

Interestingly, several detoxification genes were more highly expressed in TEN than VK7 in 2012 (and thus were filtered out of our final candidate list for VK7) (Additional file 4). Of all genes up-regulated in TEN (N = 171), the highest fold changes were for *CYP6Z3* (AGAP008217, FC = 2.55, four probes) and *CYP6P4* (AGAP002867, FC = 2.32). Both of these p450s also showed a significant increase in basal expression in VK7 from 2012 to 2013 (Figure 3) suggesting that, given their reported role in pyrethroid resistance [15,16], these enzymes may be involved in resistance in both Tengrela and VK7.

### Target-site resistance

All three *kdr* mutations (*L1014F*, *L1014S* and *N1575Y*) in the voltage gated sodium channel have been reported in *An. gambiae* s.l from south-west Burkina Faso [3,19]. Dead and surviving VK7 mosquitoes after exposure to the LT_50_ of deltamethrin were screened for each mutation to determine whether, in addition to the increases in gene expression described above, target-site resistance contributes to the strong resistance phenotype. The *1014S kdr* allele was not found in any sample. The frequency of the *1014F* allele in the 2011-2012 control samples of *An. coluzzi* was high (0.823 to 0.880) but did not differ significantly between date of collection (Fisher’s exact test, two tailed *P* value > 0.05) (Table 1). A similar frequency of 0.877 of *1014F* was observed in 2012 from Tengrela (Table 1). Samples collected in July 2013 from the different breeding sites in VK7 and Tengrela showed a similar frequency (chi-square test = 1.69, *P* = 0.43) (Table 1). The *1575Y kdr* allele had a frequency of between 0.237 and 0.355 during the collection period in both VK7 and Tengrela. No significant increase or decrease in the frequency of *N1575Y* was observed during this time (Table 1).

**Table 1:** **The frequency of the L1014F and N1575Y mutations in** ***An*** **.** ***coluzzii*** **tested in deltamethrin resistance bioassays**

**Period**	**Status**	**L**	**F**	**n (alleles)**	**(f) L1014F**	**95% CI**	**N**	**Y**	**n (alleles)**	**(f) N1575Y**	**95% CI**
***VK7***											
**Jul-11**	Control	22	102	124	0.823	0.744-0.886	80	44	124	0.355	0.276-0.442
	Dead	23	97	120	0.808	0.728-0.870	92	34	126	0.270	0.200-0.354
	Survivors	19	95	114	0.833	0.754-0.892	80	30	110	0.273	0.198-0.363
**Oct-11**	Control	27	163	190	0.858	0.801-0.901	134	56	190	0.295	0.234-0.363
	Dead	42	194	236	0.822	0.768-0.866	174	60	234	0.256	0.205-0.316
	Survivors	15	133	148	0.899*	0.839-0.939	91	55	146	0.377*	0.302-0.458
**Jun-12**	Control	25	183	208	0.880	0.828-0.918	151	55	206	0.267	0.211-0.331
	Dead	9	45	54	0.833	0.710-0.912	42	12	54	0.222	0.131-0.351
	Survivors	14	132	146	0.904	0.844-0.943	90	56	146	0.384*	0.309-0.465
**Jul-13**	Control	16	126	142	0.887	0.823-0.934	109	33	142	0.232	0.166-0.311
***Tengrela***											
**Jul-12**	Control	14	100	114	0.877	0.803-0.931	87	27	114	0.237	0.162-0.326
	Dead	18	80	98	0.816	0.725-0.887	66	32	98	0.327	0.235-0.429
	Survivors	11	81	92	0.880	0.796-0.939	66	26	92	0.283	0.194-0.386
**Jul-13**	Control	24	124	148	0.838	0.768-0.893	109	39	148	0.264	0.194-0.342

Allele frequencies with 95% CI of 1014F and 1575Y *kdr* alleles in *An. coluzzi* from Tengrela and VK7. VK7 samples are stratified according to deltamethrin exposure (unexposed control samples and mosquitoes that died or survived exposure to an approximate LT_50_). In Tengrela the exposure time was one hour. Sample sets where allele frequency is significantly associated with insecticide survival (*P* <0.05) are marked by *.

### Deltamethrin resistance and *kdr* haplotype association

**Figure 5:**
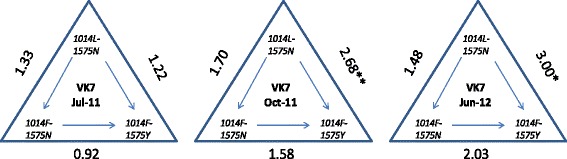
**Haplotypic association tests for the three** ***kdr*** **haplotypes (** ***1014L-1575N*** **,** ***1014F-1575N*** **and** ***1014 F-1575Y*** **) with survival of VK7** ***An. coluzzi*** **to deltamethrin LT**_**50**_ **.** Odds ratios (OR) are represented with the level of significance and the arrows within the triangles show the direction of the OR calculation. *p < 0.05, **p < 0.01.

In almost all cases the frequencies of *1014F* and *1575Y* were higher in mosquitoes surviving insecticide exposure but the presence of the *1014F* allele was only significantly associated with deltamethrin survival in one round (October 2011) (*P* = 0.04) and in the case of *1575Y*, an association with deltamethrin survival was observed in two rounds of collection (*P* = 0.015 and 0.043 in October 2011 and June 2012 respectively (Table 1)). *1575Y* occurs exclusively on a haplotypic background of *1014F* and a stronger association of the haplotype, rather than single alleles, has been demonstrated for DDT and pyrethroid resistance in some cases [19]. Haplotypic association tests revealed that the presence of both *1014F* and *1575Y* alleles increased the OR of surviving the deltamethrin LT_50_ in VK7 from all three collection rounds but the difference was only significant for samples collected in October 2011 and in June 2012 (OR 2.68 (*P* = 0.007) and 3.00 (*P* = 0.046) respectively (Figure 5)).

## Discussion

In this study, we applied a robust microarray-approach to determine the transcriptional profile of an *An. coluzzi* population undergoing further selection on top of an already strong pyrethroid resistance phenotype. We previously documented that deltamethrin resistance ratios (the LT_50_ of the local resistant population divided by the LT_50_ susceptible strain) increased from 54 to 730 in a single calendar year from the village of VK7 in Burkina Faso [5]. By analysing transcription levels of *An. coluzzi* from VK7 in both 2011 and 2012, this provided a unique opportunity to determine whether gene expression levels were simultaneously rising with pyrethroid resistance in a single wild-caught population of *An. coluzzi* from south-west Burkina Faso.

### Candidate genes for deltamethrin resistance in VK7

According to our hypotheses of over-expression in field resistant mosquitoes compared to laboratory susceptible strains, increased expression in VK7 from 2011 to 2012, and finally, higher expression in VK7 than the more moderate resistance phenotype displayed in nearby Tengrela, we filtered microarray probes down to a final list of 157 representing 136 genes which are potentially contributing to deltamethrin resistance in VK7. These encode enzymes and proteins with both detoxification and non-detoxification functions. This gene list was compared to data generated using the same microarray platform, comparing gene expression in deltamethrin survivors of *An. coluzzi* from three independent sites in Cote d’Ivoire, with the N’Gousso susceptible strain (C Strode, unpublished data, ArrayExpress Accession numbers E-MTAB-3210, E-MTAB-3211, E-MTAB-3212). One third of the 136 genes associated with deltamethrin resistance in Burkina Faso were also found over expressed in all three populations from Côte d’Ivoire, including *CYP4G16* and the cuticular proteins *CPAP3-A1a* and *CPAP3-A1b* (Additional file 5). This suggests that similar mechanisms could underlie the pyrethroid resistance that is spreading across Western Africa.

Three P450s were present in the list of VK7 candidate probes (*CYP4G16, CYP9J5*, and *CYP9M1*). *CYP4G16* is becoming increasingly associated with pyrethroid resistance with evidence for over-expression in another major vector of the *An. gambiae* complex, *An. arabiensis* [17,20]. Although *CYP4G16* does not metabolize pyrethroids directly, it may play a role in enhancing cuticular hydrocarbon synthesis as demonstrated in members of the CYP4G family previously [21,22]. The presence of additional genes with putative roles in cuticular hydrocarbon synthesis (two elongases (AGAP003196-RA & AGAP013219-RA), 3-hydroxyacyl-coa dehydrogenase (AGAP013544-RA) and fatty acyl-CoA elongase (AGAP003195-RA)) strengthens the support for the involvement of this pathway, and *CYP4G16,* in pyrethroid resistance in *An. gambiae*, although further functional validation is needed. Furthermore, the Cuticular Proteins Analogous to Peritrophins (CPAP) class of proteins are expressed in cuticule forming tissues and maintain the structural integrity of the cuticle [23]. Two members of the CPAP3 family were consistently over-expressed in VK7 (*CPAP3-A1a* (AGAP000989-RA) and *CPAP3-A1b* AGAP000987-RA)). *CPAP3-A1b* was recently over-expressed in *An. gambiae* from an agricultural area in Northern Tanzania [24] and therefore this protein class warrants further investigation as part of any cuticular resistance hypothesis in this species.

The aquaporin (AGAP010326-RA) and chymotrypsin-1 (AGAP006709-RA) were both significantly and highly over-expressed in VK7. The precise role for these proteins in insecticide resistance remains unclear. Aquaporins are ubiquitous water transporting membrane proteins found in the Malpighian tubule system of insects [25]. The aquaporin over-transcribed in this study was also the most significantly and highly over-expressed gene in a genome-wide transcriptional profile of an extremely resistant DDT strain of *An. coluzzi* from Ghana (survival after 6 hr exposure to DDT 4%) [10]. The presence of aquaporins in the Malpighian tubules, which are also a potential site for p450-mediated metabolism [26], may represent an efflux/excretion system for insecticide detoxification. In the field of drug resistance, the loss of the activity of one of these transporters leads to resistance to melaminophenyl arsenic (MPA) and pentamidine treatment in the African Trypanosomiasis parasite (*Trypanosoma brucei*) [27]. Chymotrypsins are serine proteases secreted in the midgut of insects [28]. Chymotrypsin transfected *Culex pipiens pallens* cells treated with deltamethrin showed greater viability compared to control cells [29] but it is not clear how this enzyme class would directly or indirectly increase insecticide tolerance.

Other metabolic enzymes were present in our VK7 candidate list including two carboxylesterases (*COEAE3G*, *COEAE4G*) and a GST (*GSTE5*). The expression of two of these genes, *COEAE3G* and *GSTE5,* was greater in VK7 in 2012 according to our qPCR assays. Although the up-regulation of these enzymes has been shown in pyrethroid and DDT resistant mosquito populations elsewhere [12,13,30], it is more likely that these enzymes either play a secondary role or are involved in resistance to other compounds. For example, members of the epsilon class of GSTs, and in particular *GSTE2*, can metabolise DDT [31] and we included this in the qPCR assays given the strong evidence in its role in DDT resistance and the elevated expression in the field *An. coluzzi* from VK7 and Tengrela. The VK7 population displays intense DDT resistance with 50 hours of DDT exposure inducing only 34.3% of mortality (Toé *et al*, data not shown) and it is possible that *GSTE2* and/or *GSTE5* are involved in resistance to DDT rather than to pyrethroids [11,32].

As part of our filtering process to determine candidate genes from VK7, we decided to choose only those genes over-expressed in VK7 compared to Tengrela, however, when analysing this data we noticed that two strong candidate P450s for pyrethroid resistance, *CYP6Z3* and *CYP6P4* [15,16], were more highly expressed in Tengrela in 2012. However, by 2013, a year which saw pyrethroid resistance ratios exceed 1000× in VK7 [5], the expression of these two P450s in VK7 increased significantly, suggesting that these P450s may be contributing to the resistance phenotype in both locations.

The vast majority of transcriptional studies investigating the association of gene expression with insecticide resistance have focussed on the over-expression of candidate genes. While there is a large body of evidence to support this approach, the down-regulation of genes as part of key biological pathways should not be ignored. Additional file 6 provides a list of the 291 probes (254 genes) down-regulated in all resistant strains versus the susceptible lab colonies including those under-transcribed in VK7 compared to TEN. This list includes many transporter proteins (including odorant binding proteins) and ion channels plus a small number of cytochrome P450 genes.

### The *1014F*-*1575Ykdr* haplotype only accounts for a small portion of the resistance phenotype in VK7

The link between the *kdr 1014F* allele in the target sodium channel and the ability to survive pyrethroids or DDT is clear with selective sweeps acting upon this allele throughout different parts of Africa [7]. However, it is also evident that *kdr* alone does not account for all of the phenotypic variation in resistance. In this study, *1014F* or *1575Y* alone were only mildly associated with an increased ability to survive the LT_50_ for deltamethrin. The *1014F kdr* diagnostic might only have a limited impact in populations with high levels of pyrethroid resistance, as reported here, and elsewhere in West Africa [33]. The *N1575Y* mutation is found exclusively on a *1014F* haplotypic background and this haplotype has been shown to confer additional selective advantage in presence of insecticides [19]. The *1014F-1575Y* haplotype did provide some protection to deltamethrin (Figure 5) but the highest OR observed of 3.00 does not account for the extremely high resistance ratios observed in VK7.

## Conclusions

Using a quantitative approach to resistance monitoring we recently documented the highest level of deltamethrin resistance recorded in field-caught *An. gambiae* to date. Transcriptional profiling of these resistant mosquitoes has identified a suite of candidate genes, which can be used to monitor resistance levels as attempts are made to mitigate against development of additional resistance in the future using new vector control tools [34]. Furthermore, the findings presented here provide evidence for additional mechanisms which might be contributing to such a strong phenotype (i.e. cuticular synthesis) and urgently need further investigation.

## Methods

### Mosquito collections and genotyping

All mosquitoes used for the genotyping and microarrays were collected in June of 2011 and July of 2012 from breeding sites as larvae and reared to adults at the insectaries of CNRFP. In 2013, these included adults emerging from larvae collected in paddy fields and open puddles and reared separately. Mosquitoes were collected from two study sites of VK7 (11°39’N, 04°41’W) and Tengrela (10°40’N, 4°50’W) where the level of insecticide resistance has been characterised and its impact on LLIN efficacy reported [5]. Prior to RNA extraction, two or three legs of each individual mosquito taken from deltamethrin-treated or non-treated bioassays were removed for PCR identification and genotyping before placing the remaining body in RNA later (Sigma). Genomic DNA was extracted from mosquito legs by boiling in 20 μl 1× PCR buffer or from whole mosquitoes using Livak buffer [35]. Identification of species within the *An. gambiae* complex was performed using SINE-PCR [36]. The frequencies of the *1014F*, 1014S and 1575Y *kdr* mutations were assessed using TaqMan® PCR assays [19,37]. Genotyping was performed on all the control, surviving and dead mosquitoes exposed to the LT_50_. Allele frequencies were compared between dead and surviving insects using a 2×2 contingency table (Fisher's exact test, two-tailed *P-*value). Haplotype association tests between *1014L-1575N*, *1014F-1575N* and *1014F-1575Y* and survival to deltamethrin were performed using the Haploview software v.4.2 [38] and the odds ratio for survival and chi-square tests for association conducted for each round of insecticide testing.

### Whole genome microarray study design

All the mosquitoes used for microarrays and the qPCR assays were confirmed as *An. coluzzi* (formerly *An. gambiae* M molecular form) [39]. Two independent whole-genome microarray experiments were performed to identify candidate genes associated with deltamethrin resistance in VK7 using an interwoven a loop design (Figure 1) [40]. The interwoven loop design used in our experiments has been shown to provide more power in detecting small differences in gene expression [41]. The first experiment compared VK7 mosquitoes collected in 2011 that were a) unexposed to insecticides (VKC) and b) had survived a 4 hr exposure to 0.05% deltamethrin (VKR) with an insecticide susceptible strain from Mali (MAL). In the second experiment, VK7 mosquitoes collected in the following summer in 2012 which had survived a 10 hr exposure to deltamethrin (0.05%) (VKR) were compared to MAL and a second fully susceptible strain from N’Gousso (NG) (Cameroon). In this second experiment, an unexposed population from Tengrela (TEN), a rice field area located approximately 120 km from VK7 which showed 50% mortality following one hour exposure to deltamethrin, was added to the design. The objective of this expanded study in 2012 was to improve the confidence in our candidate gene list by including a second susceptible strain and an additional field strain with a less intense resistance phenotype. Each biological replicate in the microarray consisted of ten non-blood females and were 5 days old.

### RNA extraction and microarray data analysis

Mosquitoes exposed to insecticide were retained for a further 24 hours prior to storage in RNA later (Sigma) to minimise any induction effect from insecticide exposure and unexposed mosquitoes were exposed to untreated control papers. Total RNA was extracted from pools of ten mosquitoes using RNAqueous®-4PCR Kit for isolation of DNAse-free RNA (Ambion) according to the manufacturer’s protocol. The quality and quantity of the RNA used for the microarrays was assessed using a Nanodrop spectrophotometer (Nanodrop Technologies) and 2100 Bioanalyser (Agilent Technologies). cRNA (100 ng) was synthesised and labelled with cyanine 5 (cy5) or cyanine 3 (cy3) using the Two-Color Low Input Quick Amp Labelling Kit (Agilent Technologies) according to the manufacturer’s instructions. Labelled cRNA were then column purified (QIAGEN) and eluted in 30 μl of RNase free water. Labelled cRNA samples (300 ng) were hybridized to the 8×15K Aligent whole-transcriptome *An. gambiae* microarray chip (A-MEXP-2196) [10] for 17 h at 65°C. Slide washing, scanning and feature extraction were performed as described previously [10].

The raw array data were normalised using LIMMA [42] prior to applying a MAANOVA model in R software [43]. The 2011 and 2012 results were combined and a hypothesis driven filtering approach of the probe sets applied to identify candidate genes in VK7. A schema of our approach is given in Figure 2. Under our hypothesis of greater expression in resistant mosquitoes, genes were considered candidates if probes were significant (false discovery rate adjusted *P* value (q) < 0.05) and the fold-changes were consistently up-regulated in all pair-wise comparisons between resistant versus susceptible mosquitoes (Steps A-C, Figure 2). This provided a set of baseline candidate probes from which probes were filtered further if they were significantly over-expressed (q < 0.05) in VK7 compared to TEN (Step D, Figure 2) and showed greater expression in 2012 compared to 2011 (Step E, Figure 2).

### qPCR for candidate gene expression in VK7 and TEN

The expression of a selection of nine genes from the microarray analyses was validated using reverse-transcription quantitative PCR (RT-qPCR). All mosquito samples were unexposed to insecticides. Each replicate was independent of the samples used in the microarray to provide external validation of the expression data. Our hypothesis was based on the rapid rise of resistance in VK7 during the time period between June 2011 and July 2013 and therefore we anticipated expression of candidates to concurrently rise. According to this hypothesis we were concerned primarily with the changes in expression levels in the resistant sites rather than pair wise comparisons with susceptible strains. As a result only samples from VK7 and Tengrela were tested in the qPCR. Primers were designed using PrimerBLAST tool (NCBI: http://www.ncbi.nlm.nih.gov/tools/primer-blast/) against the *An. gambiae* PEST sequence and at least two primer sets were used to assess their efficiency and specificity by running a standard curve over a five-fold serial dilution (Additional file 7). Approximately 600 ng of RNA was reverse transcribed to first strand cDNA using SuperScript™ III reverse transcriptase (Invitrogen). All six biological replicates for each population were run in triplicate using 5 μl of input cDNA diluted 10-fold, 2× SYBR Brilliant III (Aligent Technologies) and 300 nM of the forward and reverse primer on the Mx3005P qPCR system (Aligent Technologies). The thermal profile for each reaction was 95°C for 3 min followed by 40 cycles of 95°C for 10 s and 60°C for 10 s. The qPCR data were analysed according to the ddCt method [18] relative to the average of three housekeeping genes encoding a ubiquitin protein (AGAP007927), an elongation factor (AGAP005128) and the S7 ribosomal protein (AGAP010592). The qPCR efficiency of each primer set was incorporated into the reaction. The Ct data were log-transformed to ensure a normal distribution of the data prior to statistical analysis. To determine whether a significant changes in expression levels occurred during the timeframe of the study (in line with increases in deltamethrin resistance), normalised mRNA levels were compared between each time point for each population separately using a two-tailed *t*-test (*P* <0.05).

### Supporting data

The data sets supporting the results of this article are included within the article and as additional files. Microarray data are available in the ArrayExpress database (www.ebi.ac.uk/arrayexpress) under accession numbers E-MTAB-2859 and E-MTAB-2875.

## Additional files

Additional file 1:
**List of 605 probes up-regulated in field resistant populations versus laboratory susceptible strains (steps A, B & C of Figure** 2 **schema).** Probes listed in order of fold change between the VK7/MAL comparison in 2012 (highlighted in grey). Additional file 2:
**List of 374 probes up-regulated in field resistant populations versus laboratory susceptible strains and showing higher expression in 2012 than 2011 (steps A, B, C & E of Figure** 2 **schema).** Probes listed in order of fold change difference between the VK7/MAL comparison in 2012 versus 2011 (highlighted in grey). Additional file 3:
**List of 157 candidate probes for deltamethrin resistance in VK7.** Probes listed in order of fold change between the VK7/MAL comparison in 2012 (highlighted in grey). Additional file 4:
**List of probes showing over-expression in Tengrela compared to VK7 in 2012.** Probes listed in order of fold change between TEN versus VK7 in 2012 (highlighted in grey). Additional file 5:
**Over-expressed genes from three populations of** ***An. coluzzi*** **from Côte d’Ivoire associated with deltamethrin resistance that are also up-regulated in VK7 in this study.** The fold-changes for three populations (Bouaké, M’Be and Tiassalé) compared to N’Gousso are listed with the transcript ID and description. Additional file 6:
**List of 271 probes significantly down-regulated in resistant populations versus laboratory susceptible strains and under-expressed in VK7 versus TEN.** Probes listed in order of the most under-expressed probes between the VK7/MAL comparison in 2012 (highlighted in grey). Additional file 7:
**Information on primers used for qPCR validation of candidate genes.**
